# The Effect of Caffeine Exposure on Sleep Patterns in Zebrafish Larvae and Its Underlying Mechanism

**DOI:** 10.3390/clockssleep6040048

**Published:** 2024-11-18

**Authors:** Yuanzheng Wei, Zongyu Miao, Huixin Ye, Meihui Wu, Xinru Wei, Yu Zhang, Lei Cai

**Affiliations:** 1 Guangdong Provincial Biotechnology Research Institute (Guangdong Provincial Laboratory Animals Monitoring Center), Guangzhou 510663, China; yzwei87@163.com (Y.W.); miaozy@gdlami.com (Z.M.); yhx@gdlami.com (H.Y.); wmh@gdlami.com (M.W.); 2 Guangdong Provincial Key Laboratory of Advanced Drug Delivery Systems, Guangdong Pharmaceutical University, Guangzhou 510006, China; weixinru001013@163.com

**Keywords:** caffeine, zebrafish larvae, developmental toxicity, sleep disorder

## Abstract

The effect of caffeine on the behavior and sleep patterns of zebrafish larvae, as well as its underlying mechanisms, has been a topic of great interest. This study aimed to investigate the impact of caffeine on zebrafish larval sleep/wake behavior and the expression of key regulatory genes such as cAMP-response element binding protein (CREB) and adenosine (ADA) in the sleep pathway. To begin, the study determined the optimal dose and duration of caffeine exposure, with the optimal doses found to be 31.25 μM, 62.5 μM, and 120 μM. Similarly, the optimal exposure time was established as no more than 120 h, ensuring a mortality rate of less than 10%. The confirmation of these conditions was achieved through the assessment of angiogenesis and the inflammatory reaction. As a result, the treatment time point of 24 h post-fertilization (hpf) was selected to examine the effects of caffeine on zebrafish larval sleep rhythm (48 h, with a light cycle of 14:10). Furthermore, the study analyzed the expression of clock genes (bmal1a, per1b, per2, per3, cry2), adenosine receptor genes (adora1a, adora1b, adora2aa, adora2ab, adora2b), and key regulatory factors (CREB and ADA). The research confirmed that caffeine could induce sleep pattern disorders, significantly upregulate adenosine receptor genes (adora1a, adora1b, adora2a, adora2ab, adora2b) (*p* < 0.05), and markedly decrease the total sleep time and sleep efficiency of the larvae. Additionally, the activity of ADA significantly increased during the exposure (*p* < 0.001), and the tissue-specific expression of CREB was also significantly increased, as assessed by immunofluorescence. Caffeine may regulate circadian clock genes through the ADA/ADORA/CREB pathway. These findings not only enhance our understanding of the effects of caffeine on zebrafish larvae but also provide valuable insights into the potential impact of caffeine on human behavior and sleep.

## 1. Introduction

Sleep is a fundamental and unavoidable requirement for animal life [1]. In recent years, a growing body of research has revealed that the primary function(s) of sleep are conserved [1,2,3,4], and this progress is largely owed to animal models including zebrafish. Interestingly, in contrast to the nocturnal rhythms of rodents (rats or mice), zebrafish sleep rhythms are similar to humans as well as diurnal in nature [5]. The pineal gland of zebrafish is fully formed at 19–20 h post-fertilization (hpf) [6], and melatonin is produced by it at night under the control of the circadian clock [6,7]. This species of larvae can exhibit complex sleep behaviors as early as 4 d post-fertilization (dpf) [3,8,9]; moreover, small body size can be tracked by videography in a 96-well plate format. Considering that the conservation of brain regions, with more than 70% of homologous genes compared to humans [10], zebrafish are an excellent model organism for studying the genetic and neural control of sleep biological processes. Furthermore, zebrafish have emerged as a powerful model for lead compound screening and assessment [11,12].

Caffeine is one of the most common naturally occurring psychoactive stimulant drugs in use worldwide, and it has a robust effect on the circadian period of molecular oscillations [13,14]. Caffeine acts as an adenosine receptor antagonist and exerts its effect in a dose-dependent manner [15]. Anecdotal evidence suggests that high concentration of caffeine can cause insomnia, dizziness, and anxiety [16,17]. Its effects of increasing alertness and decreasing fatigue and drowsiness were also confirmed in many investigations on zebrafish [17,18,19,20]. Even though zebrafish research has made rapid progress in understanding the development and function of sleep circuits, studies based on caffeine-induced sleep disorder models in zebrafish larvae have not progressed as swiftly. The main reason is, for one thing, the locomotor behavior of zebrafish has exhibited a completely different response: low to medium doses increase activity, but high doses decrease it [15]. For another, the “light–dark locomotion test” is based on the adult zebrafish, and most current studies focus on the caffeine-induced anxiety-like behavior; furthermore, larval zebrafish are more commonly used in embryonic developmental neurotoxicity [21,22,23]. Only a very few studies have explored the effects of caffeine on the sleep rhythms in larval zebrafish at 6 dpf [24] and 5 dpf [25]. Thus, why the circadian system is sensitive to caffeine has remained a mystery, and the effects of caffeine on the formation of sleep homeostasis of zebrafish larvae have not been demonstrated unequivocally. Meanwhile, it has been demonstrated that caffeine treatment induces defects of angiogenesis in zebrafish embryos [26]. High concentrations of caffeine would cause developmental toxicity, and skeletal muscles appear smaller and lack [27]. Caffeine reduces hepatic lipid accumulation through the regulation of lipogenesis and ER stress in zebrafish larvae [28]. All these pose more challenges to the successful construction of sleep disorder models in the larval stage; moreover, the “optimum test time” (5 dpf~7 dpf) must be considered in the specific experiment. Not only is the relatively perfect sleep rhythm of larvae formed in 4 days, but they also usually need to be fed after 7 days. If caffeine exposure is done before 4 dpf of embryo development, it can avoid the effects of caffeine on Chinese herbal medicine noticeably. Therefore, it is of practical significance to investigate the effects of caffeine on sleep rhythm formation in the early life stages of zebrafish.

The aim of this study was to evaluate how caffeine administration is suitable for building zebrafish sleep disorder models in the early life stages. Considering that angiogenesis and inflammation are mutually dependent [29] and changes in the immune system can also alter the sleep architecture [30], caffeine exposure time and concentrations on angiogenesis and inflammatory response embryonic development were investigated. Moreover, focusing on the effects of caffeine on the circadian rhythm and neurochemistry, we further explored the effect of caffeine on the adenosine system in the larvae. These results will have practical significance for the construction of a drug-induced sleep disorder model and will help us to further understand the molecular mechanisms of sleep/wake behaviors in zebrafish.

## 2. Results

### 2.1. Toxicity of Caffeine on Developing

**Figure 1:**
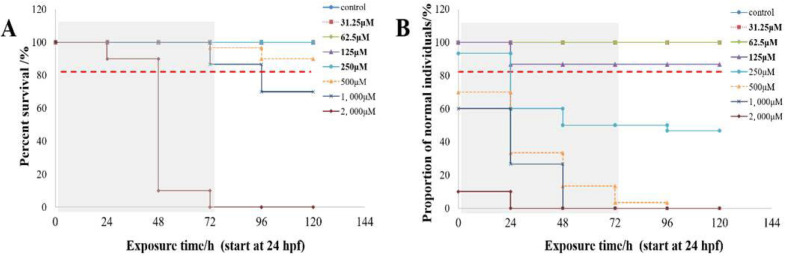
Percent survival (**A**) and proportion of normal individuals (**B**) of developing zebrafish embryos after exposure to caffeine. The red horizontal line indicates the threshold when the proportion is 80%. The gray shading marks the time before sleep rhythms were established. Each treatment group’s (31.25 μM, 62.5 μM, 125 μM, 250 μM, 500 μM, 1000 μM, 2000 μM caffeine) survival was assessed, and the number of normal individuals was counted (3 trials, *n* = 30).

Percent survival and proportion of normal individuals of developing zebrafish embryos after exposure to caffeine are shown in Figure 1. The percentage of normal individuals is represented by the absence of morphological malformations, including pericardial edema, spinal curvature, tail coiling, and so on. When 24 hpf embryos are exposed to caffeine, only 2 mM caffeine can cause significant mortality before the formation of sleep rhythms (gray shading; Figure 1A). The survival rate was more than 80% throughout the experiment when caffeine exposure did not exceed 1000 μM. Caffeine exposure for 24 h at concentrations above 125 μM showed significant deformities, and the proportion of normal individuals is less than 60% (Figure 1). The 31.25 μM, 62.5 μM, and 125 μM caffeine treatment groups showed good safety.

**Figure 2:**
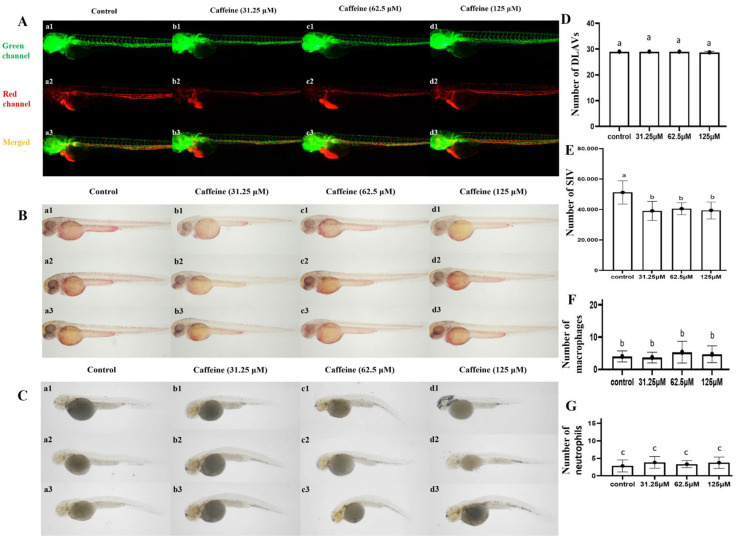
Developmental toxicity assessment of zebrafish embryos in response to caffeine exposure. (**A**) represents the development status of dorsal longitudinal anastomotic vessels (DLAVs) and subintestinal vein (SIV) after 72 h of caffeine exposure; (**B**,**C**) represent the NR labeling of macrophages and SB labeling of neutrophils in 72 h post-embryonic (hpe) caffeine-treated zebrafish larvae; (**D**) represents the DLAV numbers; (**E**) represents the SIV number; (**F**) represents the number of macrophages; (**G**) represents the number of neutrophils. The results are presented as mean ± SD (three trials, *n* = 10) and a bar chart marked with different letters indicates significant differences (*p* < 0.05). The letters a, b, and c on the bar chart represent the levels of significance. The same letter indicates no significant difference between the two, while different letters indicate a significant difference (*p* < 0.05).

To further validate the safety of zebrafish embryos exposed to caffeine at concentrations of 31.25 μM, 62.5 μM, and 125 μM, a comprehensive assessment was conducted by examining the development of the zebrafish vascular system and the induced aggregation of macrophages and neutrophils. The effects of caffeine on angiogenesis were examined at 72 hpe (hours post exposure). The results showed that the dorsal longitudinal anastomotic vessels (DLAVs) and subintestinal vein (SIV) were not affected in the 31.25 μM, 62.5 μM, and 125 μM treatments. Assessments of neutrophil and macrophage aggregation in zebrafish embryos subjected to varying caffeine concentrations of 31.25 μM, 62.5 μM, and 125 μM indicated a lack of inflammatory response under these conditions (Figure 2).

### 2.2. Sleep/Wake Behavioral Changes Induced by Caffeine in Zebrafish Larvae

**Figure 3:**
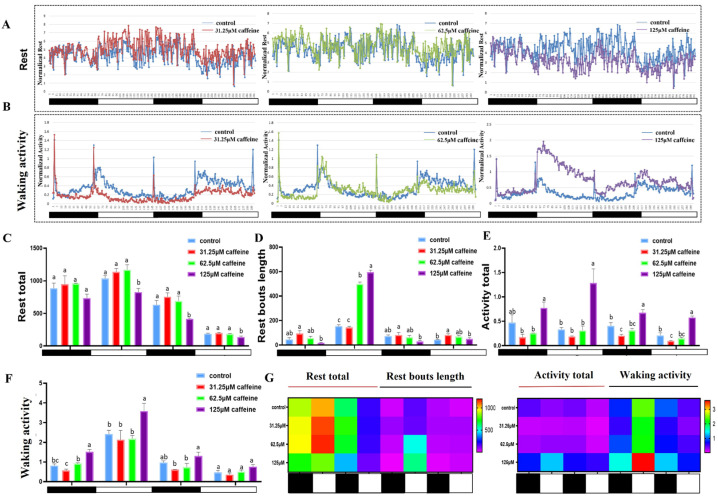
Sleep/wake behavior alterations and relevant parameters from 120 hpf to 168 hpf after caffeine was removed. (**A**) Rest total and (**B**) waking activity were averaged every 10 min and then normalized to the control values. The red lines (31.25 μM), green lines (62.5 μM), and purple lines (125 μM) show the averages of the different treated groups with caffeine, and the blue line shows the average of the control group. The black and white bars in the abscissa indicate the dark (10 h) and light (14 h) periods. (**C**–**F**) Histograms for rest total, rest bouts length, activity total, and waking activity. Values are means of quadruplicate groups and presented as mean ± standard deviation (SD, *n* = 24); main effect means that values in the same column with different superscripts are significantly different (*p* < 0.05). (**G**) Heatmap of sleep-wake related parameters. Rows indicate different behavioral parameters; columns indicate different groups. The black and white bars represente the dark and light periods respectively. The letters a, b, and c on the bar chart represent the levels of significance. The same letter indicates no significant difference between the two, while different letters indicate a significant difference (*p* < 0.05).

Sleep/wake behavior changes, assayed in the 96-well plate from 120 hpf to 168 hpf after caffeine was removed, exerted its effect in a strong dose-dependent manner (Figure 3). Rest was decreased, while wakefulness time was increased in the 125 μM caffeine treatment group compared to control at the second dark period and the two light periods (Figure 3A,B). However, the rest and wakefulness did not change significantly in the 62.5 μM caffeine treatment group, and furthermore, wakefulness was even decreased in the 31.25 μM group. Parameters including rest total, rest bout length, activity total, and waking activity were further compared among different groups. The 125 μM treatment decreased the rest time in the second dark period and the two light periods significantly (Figure 3C); meanwhile, the 31.25 μM and 62.5 μM treatments did not. Moreover, the 125 μM caffeine treatment increased the total activity and waking activity significantly (Figure 3E,F). The heatmap shows the phenotype of sleep loss and extended wakefulness (Figure 3G).

### 2.3. Caffeine Stimulates the Upregulation of Circadian Rhythm-Related Genes

**Figure 4:**
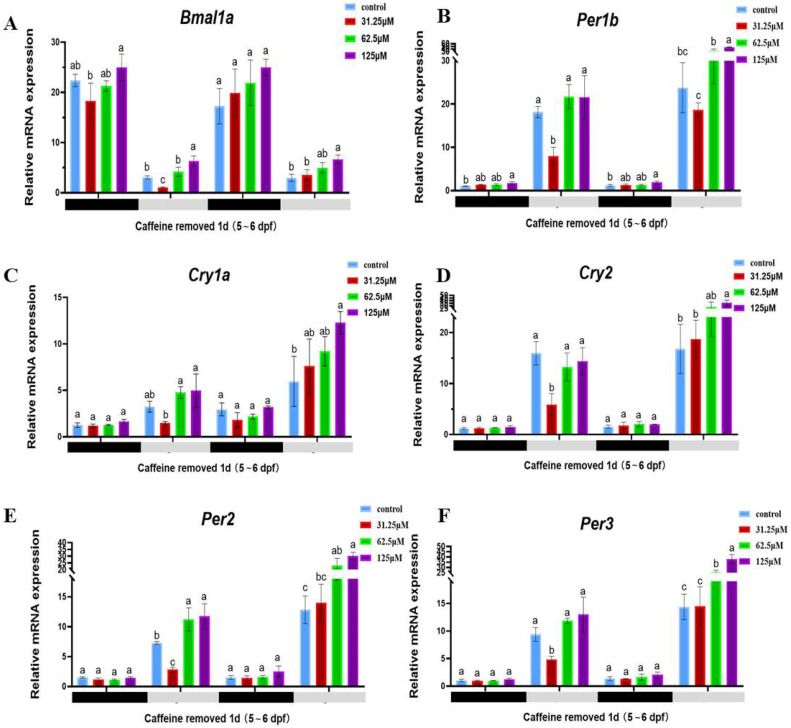
Relative mRNA expression of clock genes from 120 hpf to 168 hpf after caffeine was removed. The relative expression of each gene compared to the reference gene β-actin is shown as a bar graph. (**A**) is the *Bmal1a* gene, (**B**) is the *Per1b* gene, (**C**) is the *Cry1a* gene, (**D**) is the *Cry2* gene, (**E**) is the *Per2* gene, (**F**) is the *Per3* gene. The black bars (dark1, dark2) and light gray bars (light1, light2) in the abscissa indicate the two dark/light periods—the dark (10 h) and light (14 h) periods. The results represent the mean ± SD (*n* = 3), and a bar chart marked with different letters indicates significant differences (*p* < 0.05). The letters a, b, and c on the bar chart represent the levels of significance. The same letter indicates no significant difference between the two, while different letters indicate a significant difference (*p* < 0.05).

Corresponding to sleep/wake behavior, we also examined the expression of clock and adenosinergic receptor genes from 120 hpf to 168 hpf after caffeine was removed (Figure 4). Relative gene expressions were detected during the two dark/light periods at 139 hpf (dark1), 151 hpf (light1), 163 hpf (dark2), and 175 hpf (light2). Except for cry1a, all other five clock genes (*Bmal1a*, *per1b*, *per2*, *per3*, *cry2*) showed a significant circadian rhythm of alternating light and dark (Figure 4). Compared with the control group, high expression of clock genes was detected even at days 2 to 3 (5~6 dpf) after caffeine was removed, and it also exhibited caffeine’s effect in a strong dose-dependent manner. However, this effect was more obvious during the light periods, and only two genes, bmal1a and per1b, showed significant changes during the dark cycle.

### 2.4. Caffeine Can Induce the Up-Regulation of ADA Enzyme Activity and Adenosine Receptor Gene Expression

**Figure 5:**
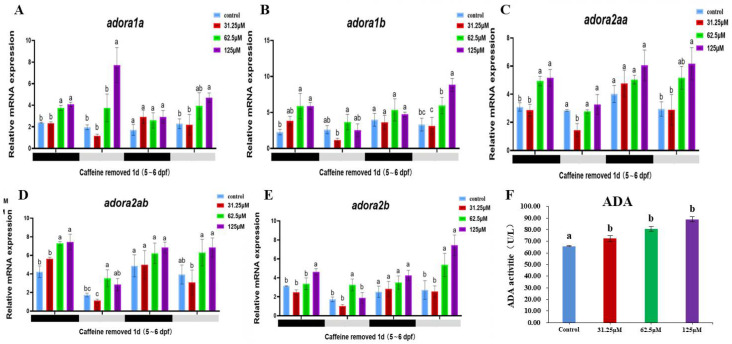
Relative mRNA expression of adenosine receptor genes from 120 hpf to 168 hpf after caffeine was removed and ADA enzyme activity in response to different concentrations of caffeine. (**A**) to (**E**) represent the diurnal expression patterns of five adenosinergic receptor genes under the influence of different concentrations of caffeine, where (**A**) is the adora1a gene, (**B**) is the adora1b gene, (**C**) is the adora2aa gene, (**D**) is the adora2ab gene, and (**E**) is the adora2b gene. (**F**) represents the enzyme activity of ADA under the influence of different concentrations of caffeine. Both the adenosinergic receptor genes and the ADA enzyme activity show an upward trend. The letters a, b, and c on the bar chart represent the levels of significance. The same letter indicates no significant difference between the two, while different letters indicate a significant difference (*p* < 0.05).

Adenosine and adenosine receptors are important factors in the regulatory pathways of circadian clock genes. By assessing the expression profiles of adenosine receptor genes in zebrafish following caffeine exposure, we observed that all five adenosinergic receptor genes (*adora1a*, *adora1b*, *adora2aa*, *adora2ab*, and *adora2b*) exhibited significant upregulation during the initial dark phase subsequent to caffeine withdrawal (Figure 5). Even though the difference in adenosine receptor gene expression was no longer significant during the second dark period, it persisted during the second light period.

Further investigation was conducted using the ELISA method to assess the activity of adenosine deaminase (ADA), the rate-limiting enzyme in the biosynthesis of adenosine. The findings revealed a pronounced positive correlation between ADA activity and escalating caffeine concentrations (Figure 5). Remarkably, at a caffeine concentration of 125 μM, ADA activity peaked, demonstrating a statistically significant difference from the control group (*p* < 0.001). Intriguingly, even at minimal caffeine concentrations, there was a substantial increase in ADA activity, thereby substantiating caffeine’s stimulatory influence on ADA.

### 2.5. Caffeine May Regulate Circadian Clock Genes Through the ADA/ADORA/CREB Pathway

**Figure 6:**
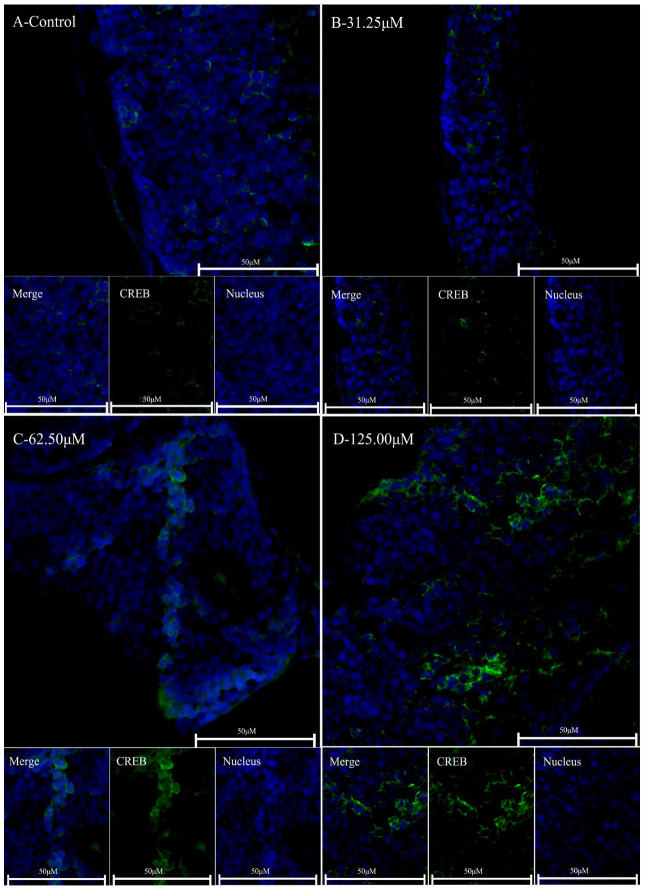
Immunofluorescence analysis of CREB in zebrafish brain tissue following exposure to varying concentrations of caffeine. (**A**) is designated as the control cohort, while (**B**–**D**) correspond to the experimental groups treated with caffeine at concentrations of 31.25 μM, 62.5 μM, and 125 μM, respectively. The term “Nucleus” refers to the cellular nuclear staining visualized in blue, “CREB” denotes the immunofluorescent labeling of the CREB protein in green, and “Merge” illustrates the composite image where the blue and green channels are superimposed.

cAMP-response element binding protein (CREB) is a downstream regulatory target gene of adenosine receptors involved in sleep regulation. Using immunofluorescence, we assessed the expression levels of CREB in the brains of zebrafish (Figure 6). Our findings indicate that caffeine administration is associated with a significant upregulation of CREB expression in zebrafish brain tissue, demonstrating a dose-dependent response. This is consistent with the results of previous studies. Previous studies have shown that zebrafish express adenosine receptor subtypes (*A1*, *A2A1*, *A2A2*, and *A2B*) since 24 h post-fertilization (hpf) and that caffeine exposure is able to affect the expression of these receptors [31]. Specifically, the groups exposed to higher concentrations of caffeine (62.5 μM and 125 μM) exhibited enhanced fluorescence intensity within the brain tissue. These results suggest that caffeine may perturb the sleep-wake cycle in zebrafish via the ADA/adenosine/adenosine receptor/CREB signaling pathway.

## 3. Discussion

Considering that high doses of caffeine have significant toxicity to embryonic development [27,32,33,34], we first focused on the optimal exposure time and concentration of caffeine administration. In this study, we also exposed embryos to caffeine at 24, 48, and 72 hpf. The experimental setup and detection interval were exactly the same as described above, except for the embryonic development period at the beginning of the experiment. When exposure time started at 4 hpf, almost all concentrations of caffeine treatments had less than 80% normal phenotypes at 96 hpf. Similar results showed that about 80% of the survival rate of zebrafish embryos was observed after exposure to 10 μM and 100 μM caffeine starting at 2 hpf [32]. Noticeably low morphologic scores (notochord and heart) were also confirmed in high caffeine concentrations (250 and 500 μM) after exposing fertilized eggs to caffeine [35]. This suggests that a high concentration of caffeine can cause significant developmental toxicity at the start of early-life stages. In view of the fact that deformities can have a serious impact on the measurement of behavioral assays [27], the optimal exposure time of caffeine administration was after 24 hpf.

Based on the assessment of mortality and teratogenicity rates, zebrafish exposed to caffeine concentrations of 31.25 μM, 62.5 μM, and 125 μM demonstrated good safety profiles (Figure 1). We concurrently evaluated the effects of these three caffeine concentrations on angiogenesis and inflammatory responses in zebrafish embryos, and the results also indicated a favorable safety profile.

In this research, we found that caffeine exposure in the early-life stages can severely affect the sleep rhythm. Sleep disorders still persisted, even though 125 μM of caffeine was removed for 3 days (Figure 3). This is similar to the timing of caffeine administration in some previous studies on an Alzheimer’s model with 100 μM caffeine [32]. Meanwhile, sleep/wake behavior changed from 120 hpf to 168 hpf, exhibiting caffeine’s effect in a strong dose-dependent manner. The rest and wakefulness did not change significantly in the 62.5 μM caffeine treatment group. However, only a very few studies have explored the effects of caffeine on the sleep rhythms in larval zebrafish (6 dpf [24] and 5 dpf [25]). Almost all of the caffeine administered used in these studies were different, and furthermore, behavioral activities were detected as locomotor measures such as distance traveled (cm/mm) and speed (cm/mm per minute) in short light and dark alternating conditions (10 min [21] or 15 min [36]). Other studies have demonstrated that high doses of caffeine (160, 320, 640, and 1280 μM) have the ability to inhibit locomotor activities in zebrafish [37]. Zebrafish embryos displayed a significant decrease in locomotor activity and somite lengths when exposed to 35, 75, and 150 mg/kg (>180.4 μM) of caffeine for 12 or 24 h [38]. The above locomotor activity parameters are more defined as anxiety-like behavior [18] and indirectly reflect the changes in sleep behavior to a certain extent. The sharp peaks during light-dark transitions are primarily due to significant changes in activity caused by increases or decreases in light intensity, which can also be understood as behavioral changes due to light stimulation. Corresponding to sleep/wake behavior, the expression of clock and adenosinergic receptors genes from 120 hpf to 168 hpf showed that caffeine exposure disrupts the circadian clock significantly, and furthermore, a strong dose-dependent manner was also detected, even when caffeine was removed. This suggests that even if the structural and functional consequences of these alterations to the nervous system caused by caffeine cannot be accurately investigated, early exposure disrupts neuromodulation related to the formation of sleep rhythms. At present, there are very few studies related to early-life stages. The latest research results show that daily high-dose caffeine intake affects some aspects of neuronal and glial development in the immature ovine cerebral cortex and cerebellum in the short term [39]. Caffeine intake promotes wakefulness in Drosophila, and short exposure increases sleep fragmentation with age, whereas prolonged exposure disrupts the circadian clock [40]. However, the effect of caffeine on sleep homeostasis is still controversial and not clearly established [41]. Meanwhile, the sleep/wake rhythm changes induced by caffeine in zebrafish larvae still needs a large amount of research data.

The continuous presence of caffeine and its main metabolite paraxanthine most likely triggers changes in the adenosine system, which may in turn affect sleep [41]. Adenosine is a very important neuromodulator in the central nervous system (CNS), the concentrations of which are directly linked to the energy metabolism of cells [42], playing neuroprotective roles in the regulation of sleep homeostatic mechanisms and the circadian clock [43]. The adenosinergic system of zebrafish is very similar to that observed in mammals [44,45]. Adenosine deaminase (ADA) is a purine metabolic enzyme that catalyzes the decomposition of adenosine and deoxyadenosine, which regulates intra- and extracellular adenosine levels [46], such as after sleep deprivation. Caffeine is a non-selective adenosine A1, A2A, and A2B receptor antagonist; meanwhile, its anxiogenic and arousing effects are probably mediated by A1 receptors in zebrafish [45]. The latest research suggests that not only is the A1 receptor important, but the A2A receptor also appears to be important for sleep–wake control [47]. In this study, it was confirmed that caffeine can induce the upregulation of the expression of the A1 and A2 genes in zebrafish, and the activity of the ADA enzyme showed an increasing trend with the rise in caffeine concentration. Caffeine can induce the upregulation of ADA enzyme activity and adenosine receptor gene expression. We also validated and found that the key sleep-regulating gene CREB [48], which is regulated downstream by adenosine receptors, is upregulated in the brain tissue of zebrafish. CREB is an important gene for sleep regulation that can participate in the regulation of the circadian clock genes and affect the circadian sleep rhythm [49]. The increased expression of CREB is likely due to changes in cAMP levels. In our experiment, we used qPCR to measure the expression of cAMP-dependent protein kinase catalytic subunit alpha-like (prkacaa/PKA) and adenylate cyclase 1a (adcy1a/*AC*). The results indicated that early exposure to caffeine in embryos leads to the overexpression of cAMP-related PKA and AC genes, and this increase is significantly higher both during the day and night at 5 days post-fertilization (dpf). Concurrently, qPCR results also demonstrated that *CREB* expression indeed rises at the transcriptional level. PKA primarily regulates transcription by directly phosphorylating transcription factors cAMP response element-binding protein (CREB), cAMP response element modulator (CREM), and ATF1. Phosphorylation is crucial in this process because when these proteins bind to cAMP response elements (CREs) in target genes, they can interact with the transcriptional coactivators CREB-binding protein (*CBP*) and p300 [50]. Moreover, recent research has revealed that under sleep deprivation conditions, caffeine can restore O-GlcNAc levels and improve cognitive function by activating the cAMP/PKA/CREB pathway in the brain [51]. Additionally, other studies have shown that extracts such as pterostilbene can inhibit melanin production and melanocyte dendritic development by suppressing the cAMP/PKA/CREB signaling pathway, thereby inhibiting the expression of tyrosinase (TYR), microphthalmia-associated transcription factor (MITF), Rab27A, Rab17, and gp100 [52]. Structural and functional analysis of zebrafish CREB indicates that it is a direct ortholog of mammalian CREB and is highly conserved [53]. Caffeine may regulate circadian clock genes through the ADA/ADORA/CREB pathway.

## 4. Materials and Methods

### 4.1. Fish Lines and Maintenance

All experimental procedures were approved by the Institutional Animal Care and Use Committee (IACUC) of the Guangdong Provincial Biotechnology Research Institute (Guangdong Provincial Laboratory Animals Monitoring Center) and carried out in strict accordance with the Standardized Welfare Terms for the Zebrafish Community [54]. Wild-type (AB strain) zebrafish and transgenic strains (Tg(fli1:EGFP); Tg(gata:DsRed2)) were obtained from the Guangdong Provincial Biotechnology Research Institute (Guangzhou, China). Adult zebrafish were raised in a recirculation system, and water quality parameters, including temperature, pH, ammonia/nitrogen concentration, and residual chlorine, were maintained at set values (28.0 ± 0.5 °C, 7.4~8.0, ≤0.5 mg L^−1^, and ≤0.05 mg L^−1^, respectively), and monitored weekly. The experimental photoperiod was set at 14:10 h light:dark. The procedures for adult culture and embryo collection were handled in accordance with the established protocols.

### 4.2. Determine the Optimal Dose and Duration of Caffeine Exposure

After zebrafish broodstock had spawned, fertilized embryos were collected. At 24 h post-fertilization (hpf), embryos with normal fertilization (no slow development, malformation, or death) were selected. The fertilized embryos were then randomly assigned to 24-well culture plates, with 10 embryos per well. A working solution of caffeine (Sigma Aldrich, St. Louis, MO, USA) at different concentrations (31.25 μM, 62.5 μM, 125 μM, 250 μM, 500 μM, 1000 μM, and 2000 μM) was added to each well, with three wells per concentration for biological replicates. The incubation conditions for the embryos followed an environmental temperature of 28 ± 0.5 °C and a light system with 14 h of light and 10 h of darkness. The working fluid was changed once after the embryos were out of the membrane. At various time points (24 h, 48 h, 72 h, 96 h, and 120 h), the total mortality and morphological deformities (including pericardial edema, spinal curvature, and tail coiling) were observed and recorded using a stereomicroscope (SMZ-475t, Nikon Imaging Japan Inc., Nishioi, Shinagawa-ku, Tokyo, Japan).

The inflammatory response was detected by staining macrophages and neutrophils. With the same treatment method, 10 embryos were collected from each treatment group at 48 h, 72 h, and 96 h for neutral red staining of macrophages. Live embryos were incubated in a 2.5 μg/mL neutral red solution for 6 h at 28.5 °C in the dark. After staining, macrophage migration was observed using a stereomicroscope (SMZ-475t, Nikon Imaging Japan Inc., Nishioi, Shinagawa-ku, Tokyo, Japan). At the same time point, 10 embryos in each treatment group were collected for Sudan black staining of neutrophils. Zebrafish larvae were fixed with 4% paraformaldehyde overnight, washed with PBS 3–5 times for 10 min each, and then treated with 25%, 50%, and 70% PBS-diluted ethanol solutions. Subsequently, a 0.04% Sudan black solution was added for staining, followed by incubation in the dark for 40 min and washing with 70% ethanol. The larvae were then washed in a shaking pot at 28 °C for 1–2 h until the body color became clear. After three washes with PBS, 10 larvae from each treatment were randomly selected for photography using a stereomicroscope (SMZ-475t, Nikon Imaging Japan Inc., Nishioi, Shinagawa-ku, Tokyo, Japan).

Transgenic zebrafish embryos labeled with blood vessels and erythrocytes were obtained by pairing TG (fli1:egfp) and TG (gata:dsred2) transgenic parent fish. With the same treatment method, 30 embryos from each group were exposed to a 1 mg/mL streptomycin solution (Beijing Solibao Technology Co., Ltd., Beijing, China) at 24 h, 48 h, and 72 h and incubated at 37 °C for 10 min to remove the egg membrane. Vascular images were acquired using fluorescence microscopy (EI-3, Nikon Imaging Japan Inc., Nishioi, Shinagawa-ku, Tokyo, Japan) to evaluate the formation of dorsal longitudinal motor vessels (DLAVs) and subcutaneous veins (SIVs).

### 4.3. Sleep/Wake Behavior Test

At 24 hpf, embryos were treated with 3 concentrations of caffeine (31.25 μM, 62.5 μM, 125 μM) for 72 h and then removed. At 120 hpf, larvae were transferred to a 96-well plate, with one individual per well and twenty-four replicates per group. A chamber filled with recirculating water was used to maintain a constant temperature of 28.5 °C. The procedure for the sleep/wake behavior test was performed and analyzed, as described in [55]. In brief, a video-tracking system, Zebrabox (Viewpoint Life Sciences Inc., Ch Bates, Montreal, France), was continuously illuminated with infrared lights and illuminated with white lights to collect the 48-h sleep/wake behavior. The dark cycle ran from 14:00 to the next 0:00, and the light cycle ran from 0:00 to 14:00. The plate frame was covered with transparent film to avoid extra evaporation. The videotracking threshold parameters were set as follows: detection threshold, 40; burst, 25; freeze, 4; and bin size, 60 s.

### 4.4. Quantitative PCR

The effect of caffeine on the expression of 11 genes (clock and adenosinergic receptor genes) related to sleep/wake behaviors was determined by real-time RT-PCR (qRT-PCR), and primer sequences are shown in Table 1. At 24 hpf, embryos were treated with 3 concentrations of caffeine (31.25 μM, 62.5 μM, 125 μM) for 3 days and then removed. The larvae at 139 hpf (dark), 151 hpf (light), 163 hpf (dark), and 175 hpf (light) were collected. Total RNA was extracted using Trizol reagent (Thermo Fisher Scientific, Newton Drive, Carlsbad, CA, USA) from 20 embryos per group following the instructions and reversed to cDNA by a PrimeScript RT reagent kit (Takara Bio Inc., Dalian, China). Quantification of gene expression was performed in triplicate using a TB Green Premix Ex Taq (Tli RNaseH Plus) (Takara, Bio Inc., Dalian, China) with detection on the ABI 7500 (Eppendorf). β-actin was used as the reference gene, and relative gene expression quantification in each sample was analyzed using the 2(−ΔΔCt) method. The qRT-PCR was conducted using the following conditions: denaturing at 95 °C for 30 s, 40 cycles at 95 °C for 5 s, 60 °C for 34 s.

### 4.5. Adenosine Deaminase Assays

Adenosine deaminase (ADA) activity was determined using enzyme colorimetry (Adenosine deaminase assay kit, Nanjing Jiancheng Bioengineering Research Institute Co. LTD., Nanjing, China). At 24 hpf, embryos were treated with 3 concentrations of caffeine (31.25 μM, 62.5 μM, 125 μM) for 5 days. Larval samples were placed in 1.5 mL plastic centrifuge tubes and homogenized in 10 volumes (*v*/*w*) of 0.65% physiological saline at 0 °C using a tissue homogenizer (Ningbo Scientz Biotechnology Co., Ltd., Ningbo, China). Tissue homogenates were then centrifuged for 10 min at 3000× *g* and 4 °C, and the supernatant thus obtained was used in subsequent assays. The principle is that ADA catalyzes the hydrolysis of adenine nucleoside to produce hypoxanthine nucleoside and ammonia, thus producing ammonia for color development. ADA activity units were defined as the production of 1 μg of ammonia nitrogen per milligram of tissue protein at 37 °C with substrate for 60 min. Samples (5–10 μg protein) were added to the reaction mixture containing 50 mM sodium phosphate buffer (pH 7.0) and 50 mM sodium acetate buffer (pH 5.0) in a final volume of 200 μL and then preincubated for 10 min at 37 °C, and the reaction was initiated by the addition of substrate (adenosine) to a final concentration of 1.5 mM. After being incubated for 60 min, the reaction was stopped by the addition of 500 μL of phenol-nitroprusside reagent (50.4 mg of phenol and 0.4 mg of sodium nitroprusside/mL). Then, 500 μL of alkaline-hypochlorite reagent (sodium hypochlorite at 0.125% available chlorine, in 0.6 M NaOH) was immediately added to the reaction mixtures and vortexed. The colorimetric assay was carried out at 635 nm. All enzyme assays were performed in ten independent experiments carried out in triplicate.

### 4.6. Immunohistochemical Analysis

Zebrafish embryos with normal development within 24 h were carefully selected and subjected to different concentrations of caffeine treatment (31.25 μM, 62.5 μM, 125 μM). The treatment duration for the zebrafish larvae was 120 h, and a blank control group was included simultaneously. Zebrafish larval samples were fixed using Karnovsky fixative (Sangon Biotech (Shanghai) Co., Ltd., Shanghai, China) in a dark room at room temperature for over 24 h. Subsequently, the zebrafish tissues were embedded in paraffin using a fully automatic dehydration and embedding machine. The thickness of the tissue sections was set at 4 μm, and they were attached to adhesive glass slides. Prior to the start of the immunohistochemistry process, the slides were baked at 60 °C for 1.5 h to remove excess embedded paraffin. Then, they were rehydrated using an environmental transparency agent (Mreda Co., Ltd., Beijing, China) and graded ethanol. Following this, the slides were subjected to peroxide inactivation (3% hydrogen peroxide methanol solution) and antigen retrieval (Sangon Biotech (Shanghai) Co., Ltd., Shanghai, China). To block nonspecific binding, the same serum used as a secondary antibody source was utilized (Sangon Biotech (Shanghai) Co., Ltd., Shanghai, China). The primary antibody, anti-CREB antibody (Hangzhou Huaan Biotechnology Co. Ltd., Hangzhou, China), was diluted according to the instructions (1:50) and incubated overnight at 4 degrees Celsius. The secondary antibody (1:500), ifluor™ 488-conjugated goat anti-rabbit IgG polyclonal antibody (Hangzhou Huaan Biotechnology Co. Ltd., Hangzhou, China), was incubated at room temperature in the dark for 1 h. The blocking agent containing DAPI (Sigma Aldrich, St. Louis, MO, USA) was used for imaging, and the fluorescence image was collected using a confocal microscope. Finally, the multi-channel fluorescence images were merged for analysis.

### 4.7. Statistical Analysis

All data are expressed as means ± standard deviation (SD). Significant differences between the parameters were analyzed using a one-way ANOVA. Statistical analyses were carried out in SPSS 19.0 (IBM Corporation, Armonk, NY, USA), and the significance threshold was set at 0.05. In cases where ANOVA identified significant differences between groups, the groups contributing to that difference were identified using Tukey’s HSD.

## 5. Conclusions

In conclusion, caffeine exposure can cause irreversible developmental toxicity in zebrafish early-life stages. Meanwhile, caffeine administration (when and how) is the key to building a zebrafish sleep disorder model. Based on the angiogenesis and inflammatory response, caffeine concentrations below 125 μM would be considered safe at 24 hpf. Our results also demonstrated that caffeine’s effect was shown in a strong dose-dependent manner on the circadian rhythm, and furthermore, sleep disorder persisted in the early-life stages even after 125 μM caffeine was removed. Abnormal changes in the adenosine system can be a trigger in this process, and sleep disorder may be caused by the cAMP signaling pathway. These results will have practical significance for the construction of a drug-induced sleep disorder model and help us to further understand the molecular mechanisms of sleep/wake behaviors in zebrafish.

## Figures and Tables

**Table 1:** Primer sequences.

Locus	Sequence (3′ → 5′)	Size Range (bp)
*cry1a*	F: CCGTGGAGACCTGTGGAT R: GTGGAAGAACTGCTGGAAGAAG	136
*cry2*	F: CACCACCGCTGTCTGAATC R: TCTTCTGTTTGCTGGGGGT	114
*Bmal1a*	F: GAAGACATTACGAGGGGCCA R: AGAGGAAACCATCAGCAGCC	113
*per1b*	F: AGGAAGGCTGACAGATGATGAATG R: CCAGAGTGGGCTAAAGCGAAGTA	149
*per2*	F: ACGAGGACAAGCCAGAGGAACG R: GCACTGGCTGGTGATGGAGA	194
*per3*	F: GTTCTGGCGGAGTAATGGAG R: TGACGACGTTTTACTGGTGC	116
*adora1a*	F: GGCTGGAACAATCTGGACAAG R: AGGATGAGTGGTGGCAACA	146
*adora1b*	F: GAACAAGAAGGTGTCCAGTCAT R: CGTGAGCAGAATGGCAATGTA	196
*adora2aa*	F: ACGCCTACAGAATACGAGAGT R: CGCACCAGACCATTGACTT	160
*adora2ab*	F: CTCTTCATCGCTTGCTTTGTG R: CTGTGACGAGGCTGTTGT	121
*adora2b*	F: GCTCAAGAACGCCACCAAT R: ACCACAGACCGATGCTTATAGT	107

## Data Availability

The original contributions presented in the study are included in the article material, further inquiries can be directed to the corresponding author/s.
